# Autophagy Is Involved in the Sevoflurane Anesthesia-Induced Cognitive Dysfunction of Aged Rats

**DOI:** 10.1371/journal.pone.0153505

**Published:** 2016-04-25

**Authors:** Xiaoming Zhang, Youfa Zhou, Mingmin Xu, Gang Chen

**Affiliations:** 1 Department of Anatomy and Cell Biology, School of Medicine, Zhejiang University, Hanzhou, China; 2 Department of Anesthesiology, Sir Run Run Shaw Hospital, School of Medicine, Zhejiang University, Hangzhou, China; 3 Department of Anesthesiology, the First Hospital of Jiaxing City, Jiaxing, China; Inserm U837, FRANCE

## Abstract

Autophagy is associated with regulation of both the survival and death of neurons, and has been linked to many neurodegenerative diseases. Postoperative cognitive dysfunction is commonly observed in elderly patients following anesthesia, but the pathophysiological mechanisms are largely unexplored. Similar effects have been found in aged rats under sevoflurane anesthesia; however, the role of autophagy in sevoflurane anesthesia-induced hippocampal neuron apoptosis of older rats remains elusive. The present study was designed to investigate the effects of autophagy on the sevoflurane-induced cognitive dysfunction in aged rats, and to identify the role of autophagy in sevoflurane-induced neuron apoptosis. We used 20-month-old rats under sevoflurane anesthesia to study memory performance, neuron apoptosis, and autophagy. The results demonstrated that sevoflurane anesthesia significantly impaired memory performance and induced hippocampal neuron apoptosis. Interestingly, treatment of rapamycin, an autophagy inducer, improved the cognitive deficit observed in the aged rats under sevoflurane anesthesia by improving autophagic flux. Rapamycin treatment led to the rapid accumulation of autophagic bodies and autophagy lysosomes, decreased p62 protein levels, and increased the ratio of microtubule-associated protein light chain 3 II (LC3-II) to LC3-I in hippocampal neurons through the mTOR signaling pathway. However, administration of an autophagy inhibitor (chloroquine) attenuated the autophagic flux and increased the severity of sevoflurane anesthesia-induced neuronal apoptosis and memory impairment. These findings suggest that impaired autophagy in the hippocampal neurons of aged rats after sevoflurane anesthesia may contribute to cognitive impairment. Therefore, our findings represent a potential novel target for pro-autophagy treatments in patients with sevoflurane anesthesia-induced neurodegeneration.

## Introduction

Surgery and anesthesia have been associated with a transient or permanent decline in cognitive function, termed postoperative cognitive dysfunction (POCD). POCD more frequently occurs in elderly patients, and age is one of the main risk factors for its development. However, the etiology of POCD is largely unknown and data are scarce [1–3]. One study demonstrated that sevoflurane anesthesia could decrease the rate of neurogenesis and neuronal survival in the hippocampus, contributing to neurotoxicity and cognitive dysfunction in aged rats [4]. Furthermore, an *in vitro* study suggested that inhalation of an anesthetic could induce caspase activation and apoptosis, and increase Aβ levels, which can ultimately affect the progression of Alzheimer disease [5]. Our previous research also revealed that sevoflurane exposure in aged rats led to endoplasmic reticulum stress-induced apoptosis of the neurons, which resulted in learning and memory deficits [6]. POCD has become a major health concern, especially in aging patients, as this effect substantially delays rehabilitation. Although little is known about POCD, the maintenance of homeostasis has been shown to be important for prophylaxis [1,7], suggesting a role for autophagy.

Autophagy is a catabolic process in which misfolded or aggregated proteins, lipids, and organelles are engulfed within double-membrane vesicles in order to balance their synthesis in cells [8–9]. Under stress, cell death has two potential pathways: immediate/necrotic cell death and delayed/apoptotic cell death. As a defense response to apoptosis, autophagy is activated to maintain cellular homeostasis and promote cell survival by blocking apoptosis pathways [10–12].

In recent years, evidence has accumulated to demonstrate that upregulation of autophagy may protect against neurodegeneration [13–14]. Thus, regulation of autophagy might be a new therapeutic strategy for some neurodegenerative disorders such as Alzheimer, Huntington, and Parkinson diseases [14–15]. Although the results of our previous study with aged rats exposed to sevoflurane indicated that anesthesia-induced stress could result in alterations at the hippocampal level and cognitive deficit [6], the relationship between sevoflurane anesthesia-induced apoptosis and autophagy in the hippocampal neurons has not been elucidated. Thus, we hypothesized that autophagy could degrade misfolded proteins under sevoflurane exposure and improve the apoptotic neurodegeneration-induced cognitive impairment of aged rats.

To explore this hypothesis, the capacity for learning and memory of aged rats was tested with a Morris water maze (MWM) test following exposure to sevoflurane. Furthermore, transmission electron microscopy (TEM) was used to detect the presence of autophagic vacuoles in the hippocampal neurons. We also carried out a quantitative evaluation of the alteration of autophagy- and apoptosis-related proteins to elucidate the relationship between sevoflurane-induced apoptosis and autophagy in the hippocampal neurons of aged rats. Overall, we confirm that autophagy plays a role in sevoflurane anesthesia-induced neurodegeneration, suggesting a new potential target for treatment of POCD.

## Methods

### Animals

This study was approved by the Institutional Animal Care and Use Committee of Zhejiang University (No.201301123) under the NIH Guide for the Care and Use of Laboratory Animals. All efforts were made to minimize the number of animals used in this study. Twenty-month-old male Sprague-Dawley rats (550–750 g) used in this study were obtained from Zhejiang Academy of Medical Sciences. The rats were housed in polypropylene cages with free access to food and water, and the room temperature was maintained at 22°C on a 12-h light, 12-h dark cycle.

### Sevoflurane Exposure

Seventy-two rats were randomly divided into 6 groups (n = 12 per group): control group (CON), rapamycin group (RAP), chloroquine group (CQ), sevoflurane group (SEV), sevoflurane plus rapamycin group (SEV+RAP), sevoflurane plus chloroquine group (SEV+CQ). Rapamycin (LC Laboratories) and chloroquine (Sigma) were respectively dissolved in dimethyl sulfoxide (25 mg/mL) and further diluted in a solution containing 5% polyethylene glycol 400 and 5% Tween 80. Rats in the RAP and SEV+RAP groups were intraperitoneally injected with rapamycin (20 mg·kg^-1^·d^-1^), rats in the CQ group and SEV+CQ group were intraperitoneally injected with chloroquine (20 mg·kg^-1^·d^-1^), and the other two groups received the same dose of vehicle solution as of two days before sevoflurane exposure, which was administered daily for one week. To induce general anesthesia, rats were placed in an acrylic anesthetizing chamber with two interfaces: one was connected to a sevoflurane vaporizer and the other was connected to a multi-gas monitor one hour after intraperitoneal injection. Rats in the SEV, SEV+RAP, and SEV+CQ groups were exposed to 2% sevoflurane delivered by a humidified 30% O_2_ carrier gas for 5 h. Rats in the CON, RAP, and CQ groups were exposed to the carrier gas without sevoflurane for the same period of time. In order to ensure sufficient ventilation, 1 mL of arterial blood was sampled at the end of sevoflurane anesthesia or sham exposure via cardiac puncture from 3 rats of each group. These rats were not used for any other part of the study. pH, arterial oxygen, carbon dioxide tension, base excess, and blood glucose were analyzed using a blood gas analyzer (Kent Scientific Corp., Torrington, CT, USA).

### MWM Test

Twenty-four hours after the sevoflurane exposure, rats were subjected to the MWM test to evaluate spatial memory abilities. A round pool (diameter, 180 cm; depth, 50 cm) was filled with warm water (25°C), which was painted black to be opaque. A platform (diameter, 10 cm) was fixed in one of the four quadrants of the pool. The platform was submerged 1 cm below the water line. All rats received four training trials every day in each quadrant of the pool. In each trial, the rats were placed into the pool at a fixed position of the quadrant facing the wall of the pool. They were allowed to swim and discover the hidden platform for 120 s. If a rat could not locate the platform within 2 min, the rat was gently guided to the platform. When the rats arrived at the platform, they were allowed to stay on it for 30 s. The time taken to find the hidden platform (escape latency) was recorded. The average time of the four trials was regarded as the result for a given rat for that day. After each trial, the rat was wiped dry before being returned to its cage. On the fifth day, the platform was removed and each rat was allowed to swim in the pool for 2 min. The number of times that the rat crossed the original platform site and the time spent in the target quadrant were recorded.

### Tissue Preparation

After the MWM test, all rats were sacrificed by intraperitoneal injection of a lethal dose of pentobarbital. Three rats from each group were perfused transcardially with 200 mL normal saline followed by 300 mL of 4% paraformaldehyde. A tissue sample (~1 mm^3^) from the left hippocampus was further fixed in 2.5% glutaraldehyde for TEM observation, and the right half of the brain was fixed in 4% paraformaldehyde for a terminal deoxynucleotidyltransferase-mediated dUTP nick-end labeling (TUNEL) assay. The hippocampus of the other six rats were quickly dissected, frozen immediately in dry ice, and stored at –80°C with the left ones for protein analysis and the right ones for mRNA analysis.

### TEM Observation

The tissues from the left hippocampus were fixed with 2.5% glutaraldehyde overnight at 4°C. After three rinses with phosphate-buffered saline, the tissues were post-fixed with 1% osmium tetraoxide for 2 h. The tissues were then rinsed with distilled water, followed by a graded ethanol dehydration series ending with propylene oxide. After infiltration in a mixture of one-half propylene oxide and one-half resin, the tissues were embedded in resin. Sections (120 nm) were cut and stained with 4% uranyl acetate for 20 min and with 0.5% lead citrate for 5 min. Autophagosomes in the hippocampal neurons were observed on a TEM (PhliphsTecnai 10, Holland).

### TUNEL Assay

A TUNEL assay was performed to detect the DNA fragmentation caused by cell death in the hippocampus of aged rats. After preparation of paraffin-embedded sections (6 μm), the TUNEL staining was carried out using an *in situ* cell death detection kit (Kaiji, Nanjing, China) according to the manufacturer instructions. The nuclei of apoptotic cells were labeled brown under DAB staining. The number of TUNEL-positive cells and the total number of cells in the hippocampus were counted at 200× magnification with a Nikon Labophot 2 microscope (Nikon, Tokyo, Japan).

### Quantitative Reverse Transcription-Polymerase Chain Reaction (RT-PCR)

Total RNA was extracted from the specimens by Trizol reagent (Invitrogen, Carlsbad, CA, USA) according to the manufacturer instructions. cDNA was synthesized from 2 μg of RNA using the PrimeScript RT reagent Kit (Takara Biotechnology, Dalian, China) following the manufacturer instructions. PCR was performed on a Roche LightCycler 480 using a LightCycler 480 SYBR Green I Master Kit (Roche, Basel, Switzerland). The primers used for quantitative PCR were as follows: ACTB (5′-AGC ACA GAG CCT CGC CTT T-3′, 5′-AGG GTG AGG ATG CCT CTC TT-3′), MAP1LC3 (5′-AGC AGC ATC CAA CCA AAA TC-3′, 5′-CTG TGT CCG TTC ACC AAC AG-3′), and SQSTM1 (5′-CAC CTG TCT GAG GGC TTC TC-3′, 5′-CAC ACT CTC CCC AAC GTT CT-3′). Target gene expression was quantified using the ΔΔCt method, with the housekeeping gene beta-actin (*ACTB*) as an internal control.

### Western Blot Analysis

The hippocampus was harvested and homogenized on ice in RIPA lysis buffer (Beyotime Biotechnology, Haimen, China), containing 1× protease inhibitor cocktail (Merck, Darmstadt, Germany) for 15 min. The supernatant was collected after centrifugation at 12,600 ×*g* for 10 min, and the protein concentration was determined by the BCA kit (Beyotime Biotechnology, Haimen, China). Protein samples were subjected to 13.5% sodium dodecyl sulfate-polyacrylamide gel electrophoresis and then transferred to a nitrocellulose membrane (Whatman, Germany). After blocking with 5% skim milk for 2 h, the membrane was incubated with primary antibodies at 4°C overnight. The primary antibodies used in this study were as follows: anti-β-actin (Cell Signaling Technology, Beverly, MA, USA), anti-SQSTM1/p62 (Medical and Biological Laboratories, Nagoya-shi, Aichi, Japan), anti-LC3 (Novus Biologicals, Littleton, CO, USA), anti-caspase-3 (Cell Signaling Technology), anti-Bax (Abcam, Cambridge, UK), anti-Bcl-2 (Abcam). The membranes were then washed with Tris-buffered saline with Tween 20 and incubated with horseradish peroxidase-conjugated secondary antibodies at room temperature for 2 h. Bands were visualized with the ECL Plus western blotting detection system (PerkinElmer, USA) and the membranes were developed on a C-DiGit Blot Scanner (Li-cor Bioscience, USA). Image Studio Version 1.1 software was used for quantification of the signals.

### Statistical Analysis

Statistical analyses were conducted using GraphPad Prism (GraphPad, San Diego, CA). Changes in the escape latency in the MWM test were analyzed by ANOVA followed by Bonferroni’s post-hoc tests. Differences were evaluated using the Student’s t-test. Values of *P* < 0.05 were considered statistically significant.

## Results

### Physiological Parameters

At the end of sevoflurane anesthesia or sham exposure, arterial blood samples from 3 rats of each group were subjected to blood gas analysis. There were no significant differences in pH, PaCO_2_, PaO_2_, glucose, and SaO_2_ among the six experimental groups in the present study, and all the physiological parameters were in the normal range (Table 1, *P* > 0.05).

**Table 1:** Effects of sevoflurane exposure on physiological parameters of arterial blood gas analysis of aged rats.

	pH	PaCO_2_ (mmHg)	PaO_2_ (mmHg)	Glucose (mmol/L)	SaO_2_ (%)
**CON**	7.35±0.03	40.2±3.3	110±12	4.2±0.4	99.0±0.5
**RAP**	7.36±0.04	41.0±3.6	112±11	4.0±0.3	99.0±0.9
**CQ**	7.33±0.03	39.9±3.7	108±14	4.3±0.5	99.0±0.7
**SEV**	7.32±0.05	41.2±3.5	110±13	4.5±0.4	99.0±1.1
**SEV+RAP**	7.34±0.06	42.3±3.8	109±14	3.9±0.6	98.0±1.3
**SEV+CQ**	7.32±0.04	40.7±3.5	108±15	4.4±05	99.0±0.6

There is no significant difference in Ph, PaCO_2_, PaO_2_, Glucose and SaO_2_ among the six groups.

PaO_2_, arterial oxygentension; PaCO_2_, arterial carbon dioxide tension; SaO_2_, arterial oxygen saturation.

### Effect of Rapamycin and CQ on Sevoflurane-induced Memory Impairment

**Fig 1:**
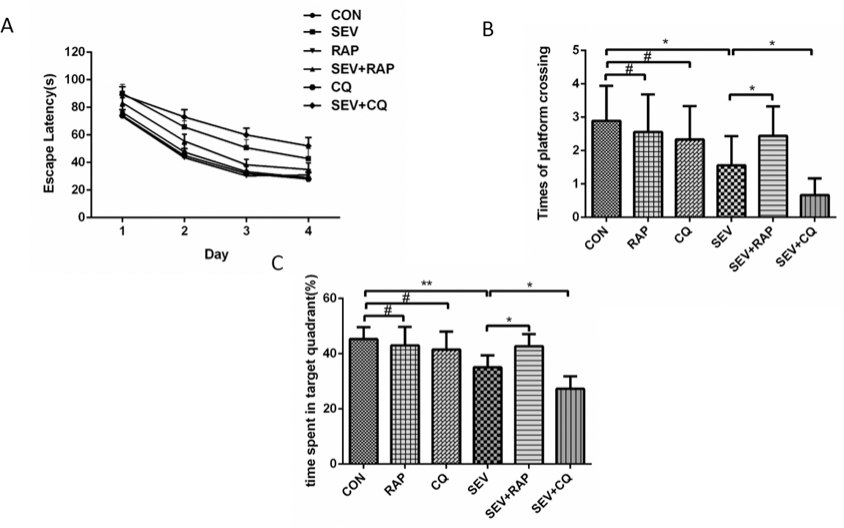
Effect of rapamycin and CQ on sevoflurane-induced memory impairment. (A) Escape latency (time to find the hidden platform) plotted against training day. Repeated measures ANOVA followed by a post hoc Bonferroni multiple comparison test: CON vs. SEV, *P* = 0.0002, *F* = 15.47, n = 9; SEV vs. SEV+RAP, *P* = 0.0155, *F* = 6.19, n = 9; SEV vs. SEV+CQ, *P* = 0.0412, *F* = 4.55, n = 9; CON vs. RAP, *P* = 0.0569, *F* = 0.33, n = 9; CON vs. CQ, *P* = 0.21, *F* = 15.47, n = 9. (B) The platform crossing times during the probe trial of the MWM test. (C) The time spent in the target quadrant during the probe test of MWM. Data are presented as mean ± SEM (n = 9). *P < 0.05, **P < 0.01, ^#^*P* > 0.05.

To evaluate the spatial learning and memory ability of aged rats from different groups, we subjected them to the Morris Water Maze. As shown in Fig 1, sevoflurane-exposed rats displayed cognitive impairment, as indicated by prolonged escape latency (Fig 1A, *P* = 0.0002, *F* = 15.47, n = 9), decreased platform crossings (Fig 1B, *P* < 0.05, n = 9) and decreased time spent in the target quadrant (Fig 1C, *P* < 0.01, n = 9), which is consistent with our previous study [6]. Importantly, the cognitive impairment induced by sevoflurane was ameliorated by rapamycin treatment, as indicated by shorter escape latency (Fig 1A, *P* = 0.0155, *F* = 6.186, n = 9), increased platform crossings (Fig 1B, *P* < 0.05, n = 9) and increased time spent in the target quadrant (Fig 1C, *P* < 0.05, n = 9). However, CQ further impaired the sevoflurane-induced cognitive impairment, as indicated by longer escape latency (Fig 1A, *P* = 0.0412, *F* = 4.547, n = 9), decreased platform crossings (Fig 1B, *P* < 0.05, n = 9) and decreased time spent in the target quadrant (Fig 1C, *P* < 0.05, n = 9). Moreover, our results showed that rapamycin and CQ alone had little effect on the cognitive function of aged rats. There was no significant difference in escape latency (Fig 1A, *P* > 0.05), platform crossings (F 1B, *P* > 0.05), and time spent in the target quadrant between the CON group and RAP group and between the CON group and CQ group.

### Confirmation of the Differences in Autophagy in the Hippocampal Neurons

**Fig 2:**
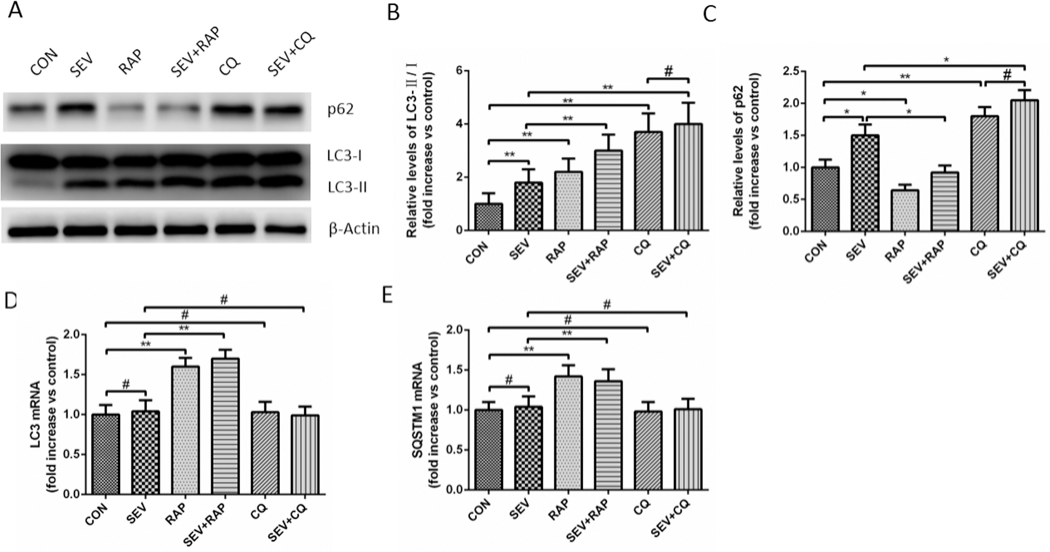
Confirmation of the differences in autophagy in the hippocampal neurons. (A) Comparison of LC3 and p62 expression in the hippocampus of aged rats. β-actin was used as an endogenous control. (B) A statistical chart of the relative optical density of p62/β-actin in each group, n = 6. (C) A statistical chart of relative optical density of LC3-II/LC3- I in each group, n = 6. (D) Relative expression of p62 mRNA in each group, n = 6. (E) Relative expression of LC3 mRNA in each group, n = 6. *P < 0.05, **P < 0.01 or ^#^ *P* >0.05.

**Fig 3:**
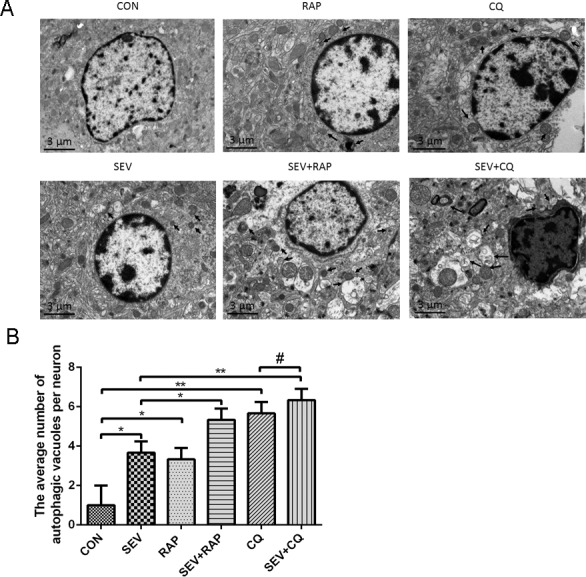
Confirmation of the differences in autophagy in the hippocampal neurons with electron microscopy. (A)Representative electron micrographs showing autophagic vacuoles in the hippocampus of aged rats in each group, n = 3. (B) Quantitative analysis of the number of autophagic vacuoles per neuron in each group. 3 rats were in each group, and ten cells were examined for each rat. The data are presented as the means±SD. Data are presented as mean ± SD. *P < 0.05 or **P < 0.01.

Conversion of LC3B-I to LC3B-II is an indicator of autophagosome formation. p62 is an adapter protein which links aggregated proteins sequestered in autophagosomes and is degraded in autolysosomes. Therefore, increase level of p62 is usually considered as indicator of impaired autophagic flux. As shown in Fig 2A, 2B and 2C, the level of LC3B-II and p62 increased significantly in rat hippocampus of SEV group compared with the CON group (*P* < 0.05), suggesting that the autophagy flux in the hippocampus of aged rats may be blocked. In addition, CQ which is classical autophagy inhibitor that blocked the process of autophagosome and lysosome fusion sevoflurane significantly increased the level of LC3 and p62 (*P* < 0.05) and when co-treated with CQ the level of LC3 and p62 was little affected by sevoflurane (*P* > 0.05), suggesting that there was intact autophagic flux in rats of CON group and impaired autophagic flux in rats of SEV group. However, on combination treatment with rapamycin, the protein expression of p62 decreased remarkably (*P* < 0.05) and the increased ratio of LC3-II/LC3-I was further enhanced (*P* < 0.05), suggesting that rapamycin partly restores the impaired autophagic flux induced by sevoflurane. The results of RT-PCR showed that sevoflurane and CQ had little effect on mRNA levels of LC3 and SQSTM1 and rapamycin increased the mRNA level of LC3 and SQSTM1 (Fig 2D and 2E). Moreover, the number of autophagosomes detected by electron microscopy in hippocampal neurons of the SEV, CQ and RAP groups was significantly increased compared to that in hippocampal neurons of CON group (Fig 3, *P* < 0.05). Significantly higher numbers of autophagosomes were detected in the SEV+RAP and SEV+CQ groups compared to that in the SEV group (Fig 3, *P* < 0.05). However, the number of autophagosomes was not significantly different between CQ group and SEV+CQ group (Fig 3, *P* > 0.05). These evidences further suggested that sevoflurane induces impaired autophagic flux in hippocampus of aged rat.

### Effect of Rapamycin and CQ on the mTOR Pathway in the Hippocampus

**Fig 4:**
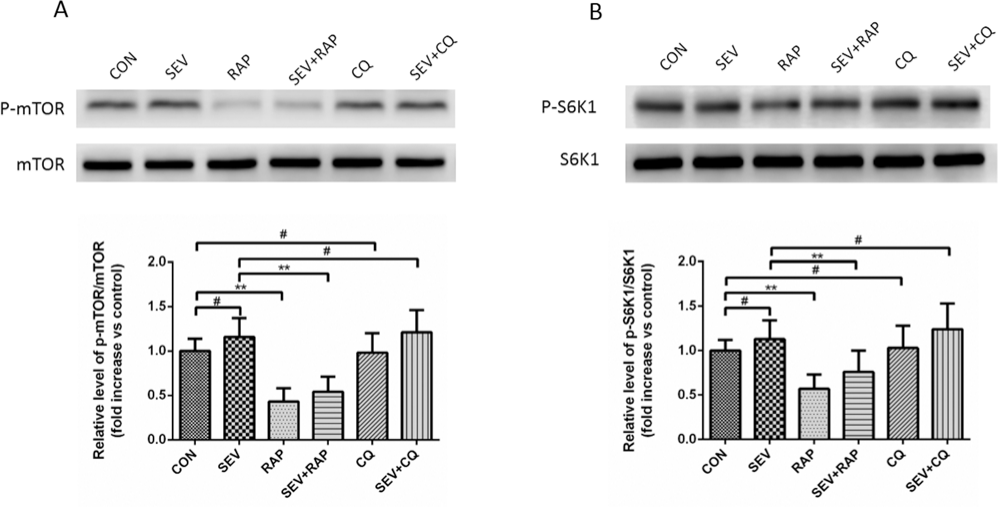
Effect of rapamycin and CQ on the mTOR pathway in the hippocampus. (A) A representative graph of western blot analysis of p-mTOR and mTOR expression in the hippocampus of each group, n = 6. (B) A representative graph of western blot analysis of p-S6K1 and S6K1 expression in the hippocampus of each group, n = 6. Data are presented as mean ± SD. *P < 0.05 or **P < 0.01, ^#^*P* > 0.05.

To investigate the mechanism of autophagy changes induced by sevoflurane, rapamycin, and CQ, we examined the mTOR activity. As shown in Fig 4, phosphorylation of mTOR and its downstream kinase S6K1 was significantly inhibited by rapamycin (*P* < 0.01), but no effect was seen in the sevoflurane and CQ groups (*P* > 0.05).

### Effect of Rapamycin and CQ on Sevoflurane-induced Apoptosis in the Hippocampus

**Fig 5:**
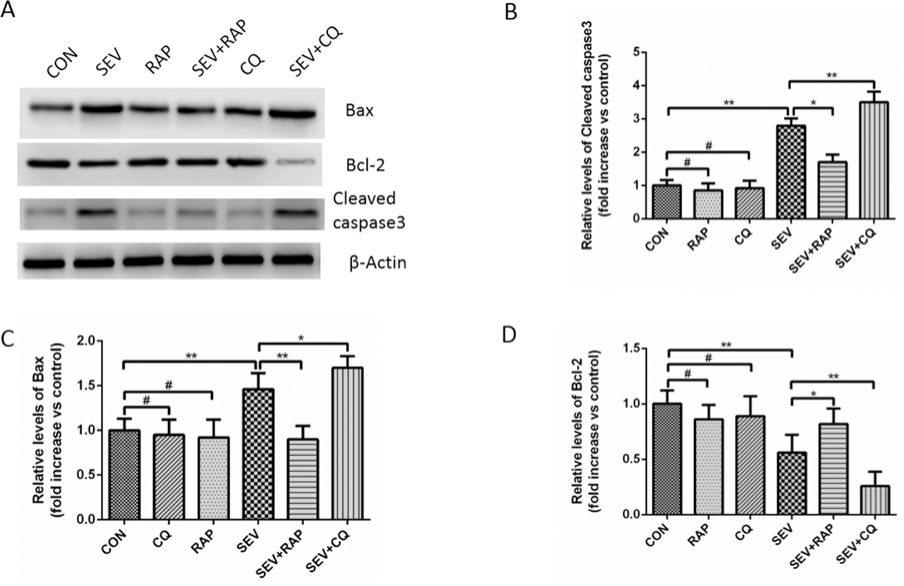
Effect of rapamycin and CQ on sevoflurane-induced apoptosis in the hippocampus. (A) Comparison of Bax, Bcl-2, and cleaved caspase3 expression in the hippocampus of aged rats in each group. β-actin was used as an endogenous control. (B) A statistical chart of the relative optical density of cleaved caspase3/β-actin in each group, n = 6. (C) A statistical chart of relative optical density of Bax/β-actin in each group, n = 6. (D) A statistical chart of relative optical density of Bcl-2/β-actin in each group, n = 6. Data are presented as mean ± SD. *P < 0.05 or **P < 0.01, ^#^*P* > 0.05.

**Fig 6:**
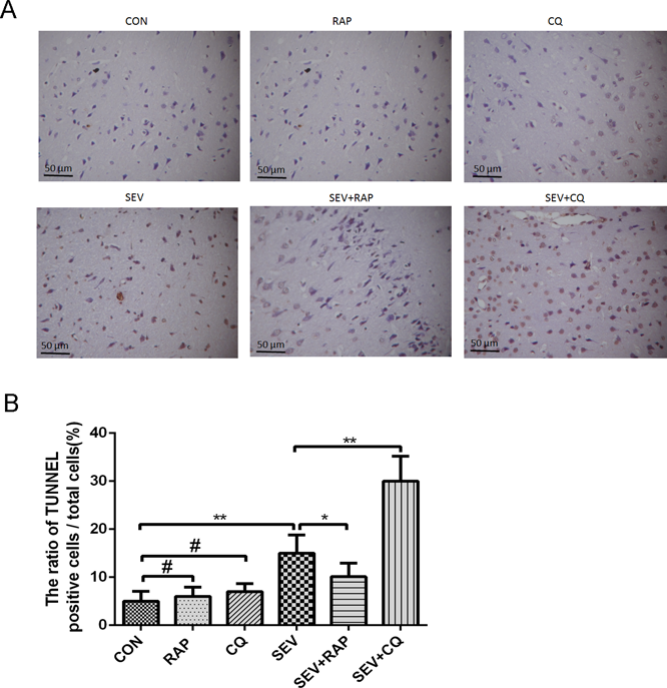
Effect of rapamycin and CQ on sevoflurane-induced apoptosis in the hippocampus analyzed by using TUNEL staining. (A) Representative photomicrographs showing TUNEL-positive cells in the hippocampus of aged rats in each group, n = 3. (B) A statistical chart of the ratio of TUNEL-positive cells/total cells, 3 rats in each group and 5 fields were observed in each rat. Data are presented as mean ± SD,*P < 0.05 or **P < 0.01, ^#^*P* > 0.05.

We examined the protein levels of cleaved caspase-3, Bax, and Bcl-2, which are well-recognized indicators of apoptosis in cells. Our results showed that sevoflurane significantly increased the expression level of cleaved caspase-3 and Bax and decreased the expression of Bcl-2 (Fig 5, *P* < 0.05). Rapamycin significantly ameliorated the increased expression of cleaved caspase-3 and Bax induced by sevoflurane and increased the expression of Bcl-2, while CQ had the opposite effect (Fig 5, *P* < 0.05). Either CQ or RAP alone has little effect on the expression of cleaved caspase-3, Bax and Bcl-2 (Fig 5, *P* > 0.05). Moreover, the results of the TUNEL assay showed that sevoflurane remarkably increased the ratio of TUNEL-positive cells/total cells in the hippocampus, CQ further increased the ratio of TUNEL-positive cells/total cells, while rapamycin decreased it. Neither CQ nor RAP has significant effect on the ratio of TUNEL-positive cells/total cells in the hippocampus when used alone (Fig 6, *P* < 0.05).

## Discussion

To our knowledge, this study is the first to show that autophagy is involved in sevoflurane-induced cognitive impairment in aging rats. There is growing concern that anesthesia may contribute to postoperative cognitive dysfunction (POCD). The incidence of POCD is higher in elderly patients under inhalational anesthesia with sevoflurane than in those maintained on intravenous propofol [2]. Over the last 60 years, many studies have shown that the hippocampus is one of the primary cognitive regions and that hippocampal formation is responsible for learning and memory formation [16]. The clinical pathological findings in humans have supported that the hippocampus is capable of neural plasticity in mild cognitive impairment [17]. Our previous research already showed that sevoflurane anesthesia could induce durable alterations and endoplasmic reticulum stress-mediated apoptotic neurodegeneration in the hippocampus of aging rats [6]. Interestingly, the present data demonstrated that the autophagy inducer rapamycin could ameliorate neuronal apoptosis and memory impairment due to sevoflurane anesthesia in aging rats. Furthermore, rapamycin treated rats showed accumulation of autophagic bodies and autophagy lysosomes in hippocampal neurons.

Autophagy is a vital catabolic process for reusing cellular components under stress conditions; however, malfunction of autophagy has been linked to a wide range of human pathologies, including cancer, pathogenic infections, and neurodegeneration [18]. The process of autophagy consists of different stages including initiation, elongation, maturation, and degradation [19]. During the process of elongation, microtubule-associated protein 1 light chain 3 (LC3), which is a mammalian ortholog of yeast ATG8, is converted from the soluble form (LC3-I) to the autophagosome-associated form (LC3-II) and plays an important role in the formation of the autophagosomes [20–21]. SQSTM1/p62, a protein associated with autophagosomes and degraded in the autolysosome, is commonly regarded as autophagy substrate protein [22–23]. As a mainly negative regulator of autophagy, activated mTORC1 inhibits autophagy induction through blocking of the initial inducer of autophagy [24–25]. Our previous results indicated that ER stress would be triggered by sevoflurane anesthesia in aging rats [6]. In response to accumulation of unfolded proteins in the ER, the ER compartment proliferates and causes damage to organelles and then induces neuron apoptosis [6]. The present study clarified that autophagy could ameliorate neuronal apoptosis and memory impairment due to sevoflurane anesthesia in aging rats. Moreover, rapamycin could increase autophagy activity by upregulation of LC3-II/LC3-I, increase in cellular autophagy vacuoles, and decrease in p62 accumulation via inhibition of the mTOR pathway. Autophagy could be activated to eliminate the irreparably misfolded proteins [26], maintain cellular homeostasis, and promote cell survival by blocking apoptosis. Furthermore, the blocking of autophagy by chloroquine could increase sevoflurane anesthesia-induced neuronal apoptosis and memory impairment.

In conclusion, the present study demonstrated that suppression of autophagy after sevoflurane anesthesia significantly impaired memory performance and induced hippocampal neuron apoptosis. Interestingly, rapamycin could ameliorate autophagic flux and cognitive deficit due to sevoflurane anesthesia in aging rats. These findings suggest that impaired autophagy in hippocampal neurons of aging rats after sevoflurane anesthesia may contribute to learning and memory impairment. Our findings provide a valid strategy for pro-autophagy treatments for sevoflurane anesthesia-induced neurodegeneration.
